# BMAL1 and CLOCK, Two Essential Components of the Circadian Clock, Are Involved in Glucose Homeostasis

**DOI:** 10.1371/journal.pbio.0020377

**Published:** 2004-11-02

**Authors:** R. Daniel Rudic, Peter McNamara, Anne-Maria Curtis, Raymond C Boston, Satchidananda Panda, John B Hogenesch, Garret A FitzGerald

**Affiliations:** **1**Center for Experimental Therapeutics, University of PennsylvaniaPhiladelphia, PennsylvaniaUnited States of America; **2**Phenomix Corporation, La Jolla, CaliforniaUnited States of America; **3**School of Veterinary Medicine, University of PennsylvaniaKennett Square, PennsylvaniaUnited States of America; **4**The Genomics Institute of the Novartis Research FoundationLa Jolla, CaliforniaUnited States of America

## Abstract

Circadian timing is generated through a unique series of autoregulatory interactions termed the molecular clock. Behavioral rhythms subject to the molecular clock are well characterized. We demonstrate a role for Bmal1 and Clock in the regulation of glucose homeostasis. Inactivation of the known clock components Bmal1 (Mop3) and Clock suppress the diurnal variation in glucose and triglycerides. Gluconeogenesis is abolished by deletion of Bmal1 and is depressed in Clock mutants, but the counterregulatory response of corticosterone and glucagon to insulin-induced hypoglycaemia is retained. Furthermore, a high-fat diet modulates carbohydrate metabolism by amplifying circadian variation in glucose tolerance and insulin sensitivity, and mutation of Clock restores the chow-fed phenotype. *Bmal1* and *Clock,* genes that function in the core molecular clock, exert profound control over recovery from insulin-induced hypoglycaemia. Furthermore, asynchronous dietary cues may modify glucose homeostasis via their interactions with peripheral molecular clocks.

## Abstract 2

Besides regulating various behavioral rhythms, the molecular clock plays a role in the control of glucose homeostasis

## Introduction

The master clock, which, in mammals, resides in the hypothalamic suprachiasmatic nucleus (SCN), is thought to synchronize multiple peripheral oscillators to ensure temporal coordination of behavior and metabolism. Peripheral clocks amplify or dampen central rhythms or exhibit autonomous behavior to facilitate local adaptive responses (Hastings et al. 2003). The central clock may communicate to modulate or entrain rhythms in the periphery via hormones (McNamara et al. 2001) or hemodynamic cues. Asynchronous environmental cues, such as eating, also influence the autonomous behavior of peripheral clocks (Damiola et al. 2000; Stokkan et al. 2001).

The variation in sleep and wakefulness (activity) is perhaps the most well-known circadian rhythm. Surgical ablation of the SCN in mice (Ibuka et al. 1980; Welsh et al. 1988) and rats (Ibuka et al. 1977; Mosko and Moore 1979) abolishes the nocturnal burst in locomotor activity. Similarly, disruption and/or mutation of Bmal1 (Bunger et al. 2000) or Clock (Vitaterna et al. 1994), transcription factors that compose the positive limb of an autoregulatory feedback loop in the core molecular clock (Young and Kay 2001; Reppert and Weaver 2002), also impairs circadian behavior. Bmal1 and Clock may influence behavioral rhythms by regulating the firing rate of SCN neurons (Herzog et al. 1998; Deboer et al. 2003).

Genes relevant to the molecular clock are also expressed in peripheral tissues (Akhtar et al. 2002; Kita et al. 2002; Panda et al. 2002; Storch et al. 2002; Oishi et al. 2003) where approximately 5%–10% of the transcriptome is subject to circadian oscillation (Albrecht and Eichele 2003). Although the precise role of peripheral clocks and the mechanisms that link them to the SCN remain largely obscure, genetic mutation or deletion has implicated peripheral clocks in the regulation of some aspects of cellular function, including division (Matsuo et al. 2003), estrous cyclicity (Miller et al. 2004), and phospholipid metabolism (Marquez et al. 2004). Glucose and lipid homeostasis are also known to exhibit circadian variation (Seaman et al. 1965; Malherbe et al. 1969; Gagliardino and Hernandez 1971; Schlierf and Dorow 1973). Surgical ablation of the SCN impairs the control of glucose homeostasis (la Fleur et al. 2001). However, the proximity of satiety centres to the SCN has potentially confounded interpretation of these results. Indeed, there is no direct evidence implicating the molecular clock in the regulation of glycaemia or insulin sensitivity (S_i_).

Our studies revealed a profound role for core clock genes—*Bmal1* and *Clock*—in regulating recovery from insulin-induced hypoglycaemia. Furthermore, the impact of a high-fat diet (HF) was to amplify the diurnal variation in glucose tolerance and S_i_ in a manner dependent on the *Clock* gene. These studies suggest that the temporal distribution of a caloric load may influence the response to insulin and that circadian variability in glucose homeostasis may be subject to modulation by asynchronous dietary cues.

## Results

**Figure 1:**
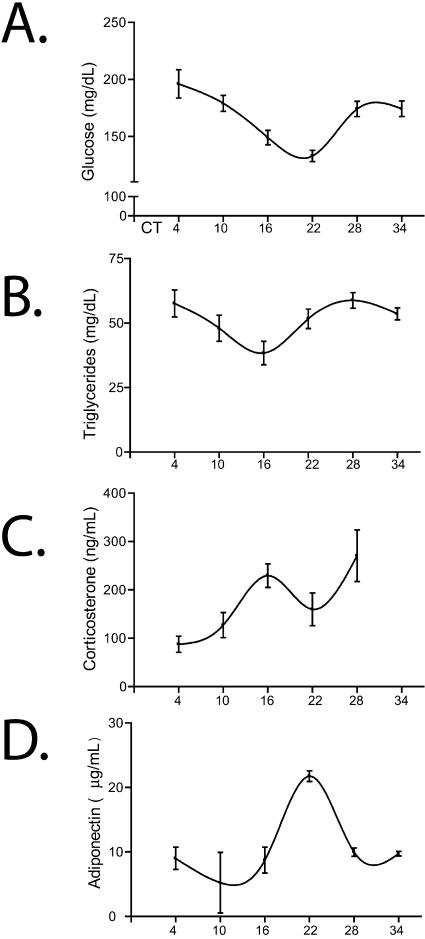
Circadian Variation of Glucose, Triglyceride, and Hormone Levels in Circulating Blood Plasma from whole blood isolated from unchallenged WT mice at different CTs was analyzed for glucose (A), triglyceride (B), corticosterone (C), and adiponectin levels (D) (*n* = 12 per time point). Results for *Bmal1* ^−/−^ and *Clock^mut^* mice are shown in Table 1.

We examined the role of the molecular clock in glucose homeostasis by using mice in which core clock genes are impaired *(Clock^mut^)* or deficient *(Bmal1^−/−^)*. Both plasma glucose and triglycerides were subject to circadian variation in wild-type (WT) mice, peaking at approximately circadian time point 4 (CT4) and CT28 (where CT0 is subjective day beginning at 7 AM, and CT12 is subjective night beginning at 7 PM) (Figure 1A and 1B), as reported previously (Seaman et al. 1965; Schlierf and Dorow 1973). We also observed that corticosterone (Figure 1C), which stimulates gluconeogenesis during hypoglycaemia (Cryer 1993), and adiponectin (Figure 1D), which has been associated with insulin resistance (Yamauchi et al. 2001; Maeda et al. 2002), oscillated significantly, but out of phase with the glucose and triglyceride rhythms. Diurnal variation in glucose and triglycerides, but not in corticosterone, was disrupted in the mutant mice (Table 1).

**Table 1:**
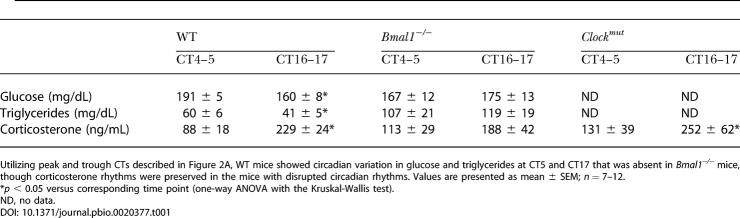
Clock-Controlled Metabolic Rhythms

**Figure 2:**
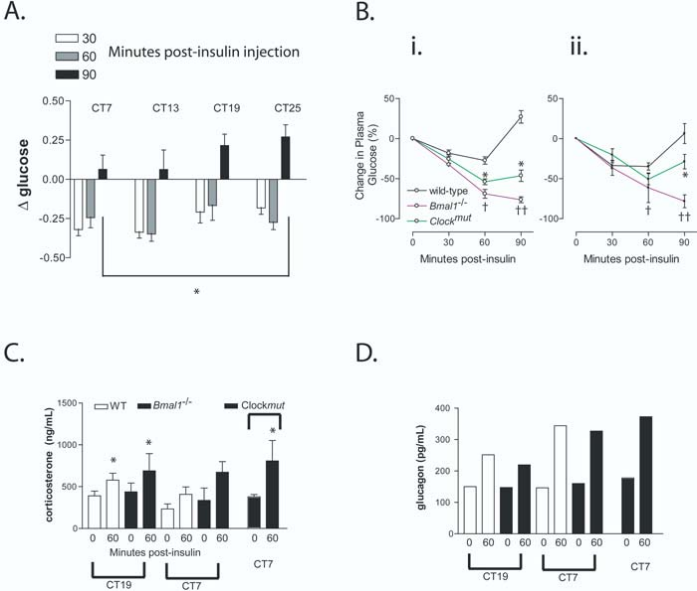
Disruption of Genes in the Core Molecular Clock Alters the Response to Insulin (A) Insulin tolerance (IT) was examined in WT mice on CT7, CT13, CT19, and CT25 at 30 min, 60 min, and 90 min after insulin injection (*n* = 12 per time point, *p < 0.01). (B) IT was examined in WT (black line), *Bmal1* ^−/−^ (blue line), and *Clock^mut^* mice (green line) at CT1 (i) and CT13 (ii) (*n* = 6–10, *p < 0.05, †*p* < 0.01, ††*p* < 0.001). (C and D) Plasma levels of the counterregulatory hormones corticosterone (C) and glucagon (D) were assessed 60 min after insulin injection in *Bmal1* ^−/−^ and *Clock^mut^* mice (*n* = 7, corticosterone assay; samples were pooled for glucagon assay, *p < 0.05).

Utilizing peak and trough CTs described in Figure 2A, WT mice showed circadian variation in glucose and triglycerides at CT5 and CT17 that was absent in *Bmal1^−/−^* mice, though corticosterone rhythms were preserved in the mice with disrupted circadian rhythms. Values are presented as mean ± SEM; *n* = 7–12

*p < 0.05 versus corresponding time point (one-way ANOVA with the Kruskal-Wallis test)

ND, no data

Although there was no clear rhythm in the hypoglyacemic response to insulin, recovery of blood glucose exhibited a robust circadian variation (Figure 2A), with an excessive rebound from the effects of insulin evident at subjective dawn (CT19 and CT25) (Figure 2A). Insulin caused a profound hypoglyacemic response, independent of clock time, in both *Bmal1* ^−/−^ and *Clock^mut^* mice (Figure 2B). This response was more pronounced in the former, consistent with the comparative severity of the molecular and behavioral phenotypes between the *Bmal1^−/−^* and *Clock^mut^* animals (King et al. 1997; Bunger et al. 2000). Despite exacerbation of the hypoglycaemic response to insulin in the mutants, the counterregulatory responses of both corticosterone and glucagon were retained (Figure 2C and 2D).

**Figure 3:**
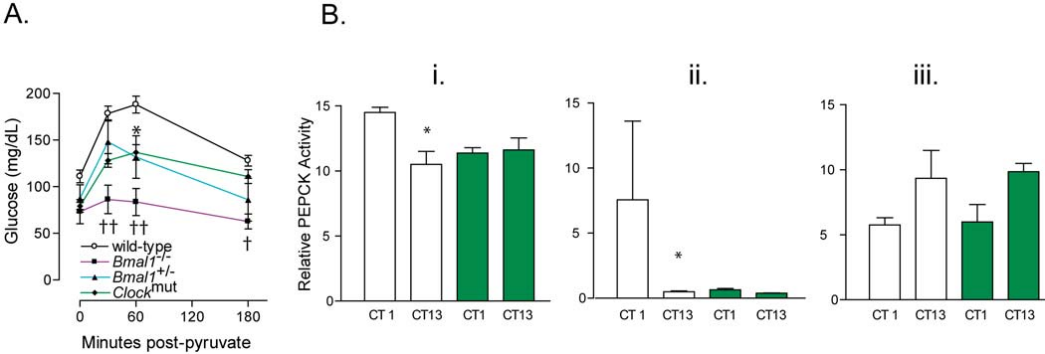
Impaired Gluconeogenesis in Mice with a Disrupted Circadian Clock (A) Pyruvate tolerance was compared among WT (black line), *Bmal1^+/−^* (blue line), and *Bmal1^−/−^* (purple line), and *Clock^mut^* (green line) mice at CT7 (*n* = 6–10). (B) Relative PEPCK activity (units are expressed as luciferase activity × 10^3^) was measured in liver (i), aorta (ii), and kidney (iii) from WT (white bars) and *Clock^mut^* mice (green bars).

Gluconeogenesis also contributes to restoration of blood glucose after insulin-induced hypoglycaemia. Consistent with this observation, conversion of exogenously administered pyruvate to glucose, which reflects gluconeogenesis (Miyake et al. 2002), was impaired in the *Clock^mut^* animals. This impairment was most marked in *Bmal1^−/−^* mice, while *Bmal1^+/−^* and *Clock^mut^* mice exhibited an intermediate phenotype when compared with WT littermate controls (Figure 3A). Furthermore, activity of the key rate-limiting enzyme of gluconeogenesis, phosphoenolpyruvate carboxykinase (PEPCK), exhibited diurnal variation in the liver and aorta that was blunted in *Clock^mut^* mice (Figure 3B). PEPCK activity in kidney was antiphasic to the rhythm in aorta and liver and was unimpaired in *Clock^mut^* mice (Figure 3B), suggesting tissue-specific regulation of enzyme activity.

The frequent sampling intravenous glucose tolerance test (FSIGT) was performed to assess more precisely the impact of the molecular clock on sensitivity to insulin. This test provides an estimate of S_i_, consistent with that obtained by the euglycaemic clamp (Pacini et al. 2001). Additionally, data modeling provides estimates of glucose-mediated glucose disposal (S_g_), insulin secretion, and S_i_. S_i_ and insulin secretion, but not S_g_, exhibited a diurnal variation in WT mice fed a regular chow diet (RC) (Table 2). Circadian variation of glucose and lipid homeostasis might condition the metabolic response to asynchronous environmental cues, such as diet, that impinge on S_i_. Dyslipidemia coincides with insulin resistance in the metabolic syndrome (Brotman and Girod 2002), and a diet high in fat impairs S_i_ (Grundleger and Thenen 1982; Coulston et al. 1983). Both HF-fed WT and HF-fed *Clock^mut^* mice increased body weight significantly and to a similar degree in comparison to their age-matched, RC-fed controls (Table 3). Body fat composition averaged 17.6% of lean body mass in RC-fed WT mice, rising to 27.7% (*p* < 0.002) on high-fat feeding. Again, fat composition was not significantly altered by the presence of the *Clock* mutation (Table 3).

**Table 2:**
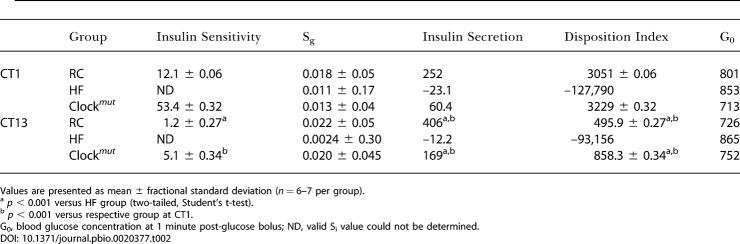
Indices of Noninsulin- and Insulin-Mediated Parameters of Glucose Disposal Derived from Modelling of FSIGT Data at CT1 and CT13

Values are presented as mean ± fractional standard deviation (*n* = 6–7 per group)

^a^ *p* < 0.001 versus HF group (two-tailed, Student's t-test)

^b^ *p* < 0.001 versus respective group at CT1

G_0_, blood glucose concentration at 1 minute post-glucose bolus; ND, valid S_i_ value could not be determined

**Table 3:**
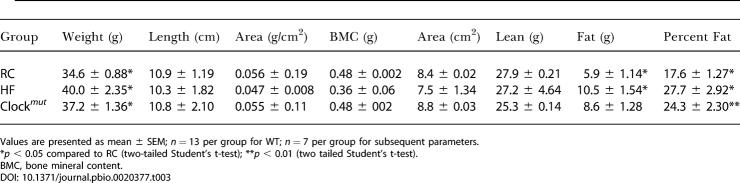
Body Mass Index Was Assessed in Mice Feeding on RC and HF

Values are presented as mean ± SEM; *n* = 13 per group for WT; *n* = 7 per group for subsequent parameters

*p < 0.05 compared to RC (two-tailed Student's t-test); **p < 0.01 (two tailed Student's t-test)

BMC, bone mineral content

**Figure 4:**
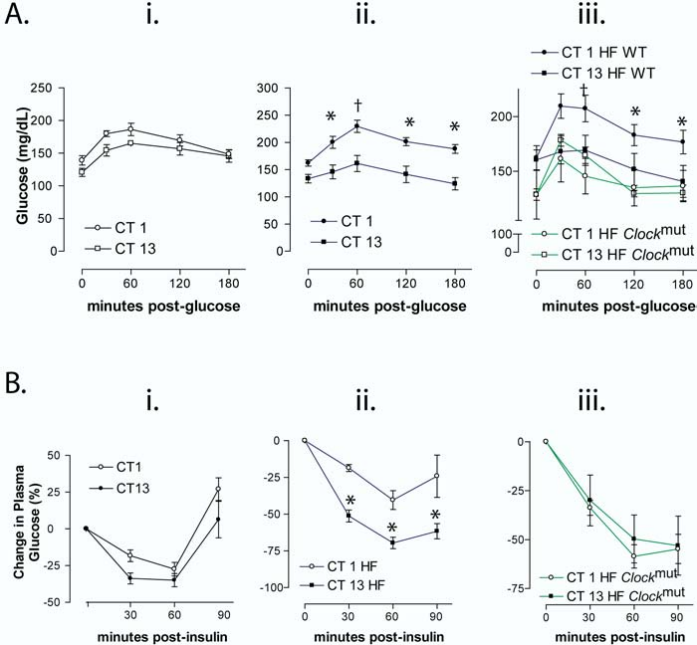
The Molecular Clock Conditions HF-Induced Circadian Variation in Glucose Homeostasis (A) Glucose tolerance (GT) in RC-fed WT mice (i), HF-fed WT mice (ii), and HF-fed *Clock^mut^* mice (iii). (B) IT in RC-fed WT mice (i), HF-fed WT mice (ii), and HF-fed *Clock^mut^* mice (iii) at respective times (*n* = 6–8; *p < 0.05, †*p* < 0.01).

Glucose tolerance on an RC trended towards an intolerant phenotype at CT1 versus CT13, but this difference did not attain significance (Figure 4A). This is consistent with the temporal variation in insulin secretion observed in RC-fed WT mice in the FSIGT experiment (Table 2). However, when the mice were fed HF for 2 mo, this glycaemic excursion at CT1 evoked by the environmental challenge was amplified and significant (two way analysis of variance [ANOVA]; *F* = 63.2, *p* < 0.001) (Figure 4A). Similarly, although the hypoglycaemic response to insulin was not different in mice fed regular chow at CT1 and CT13 (Figure 4B), the HF induced a significant temporal variation (Figure 4B). Thus, the impact of a high fat intake on carbohydrate metabolism in WTs includes an amplification of the diurnal variation in the response to both glucose and insulin. This coincides with a modest impairment in the ability to restore euglycaemia after insulin. A similar impairment resulting from a defect in gluconeogenesis has been observed in rats (Oakes et al. 1997). The mutants failed to exhibit a significant time-dependent variation in their response to glucose or insulin, again reminiscent of the RC-fed, WT phenotype (Figure 4).

Mice were also subjected to extended high-fat feeding (11 mo versus 2 mo). Long-term hyperlipidemia is known to induce frank diabetes with impaired release of insulin (Johnson et al. 1990), in contrast to short-term, high-fat feeding, which increases release of insulin, but impairs the response to it (Linn et al. 1999). The extended HF impaired insulin secretion, reflected by its marked reduction to negative values in HF-fed WT mice (Table 2). The lack of insulin secretion resulted in calculated values of S_i_ that were imperceptibly low (see Material and Methods). However, S_g_, insulin secretion, and S_i_ were restored to a WT phenotype in *Clock^mut^* mice that were also HF-fed for 11 mo (Table 2). This is, again, consistent with a role for the molecular clock in conditioning the response of glucose metabolism to the intake of dietary fat. Remarkably, mutation of the molecular clock protected against the development of frank diabetes caused by chronic high-fat feeding.

## Discussion

Maintenance of blood glucose levels within a narrow range is critical to mammalian survival, and environmental cues can trigger appropriate tissue disposition of glucose through adaptive behaviors such as in hibernation (Castex and Hoo-Paris 1987) or the “fight-or-flight” response (Surwit et al. 1992). In this sense, glucose regulation is a fundamental and ancestral defence mechanism. Our studies suggest that Bmal1 and Clock, core components of the molecular clock, contribute substantially to regulation of recovery from the hypoglycaemic response to insulin. However, other mechanisms also impinge on this ancient adaptive response. Thus, impaired recovery from insulin-induced hypoglycaemia is observed in mice lacking proopiomelanocortin. These animals lack adrenal glands and melanocortins and exhibit a defective glucagon response to insulin-induced hypoglycaemia (Hochgeschwender et al. 2003). They contrast with the *Bmal1* ^−/−^ and *Clock^mut^* mice, where the counterregulatory hormone response is unimpaired. Thus, steroids, epinephrine, and glucagon appear to facilitate recovery from insulin-induced hypoglycaemia in a manner distinct from, but complementary to, the molecular clock.

An assumption intrinsic to our studies is that the phenotypes revealed in the mutant mice are attributable to their function as core elements of the molecular clock. However, as trans-activators, both Clock and Bmal1 may have pleiotropic effects independent of the circadian clock that could impinge on metabolism. Several lines of evidence argue against this hypothesis. First, genes relevant to these metabolic phenotypes display circadian oscillations in their steady-state mRNA levels (Young et al. 2001; Oishi et al. 2003). In addition, the mRNA levels of many of these key proteins are phase-aligned with *Per1* (e.g., *Enolase 3, Pgam, Transketolase, Lipase, Lpl, Dgat1, Ppar alpha*) or *Per2* (e.g., *Mod1, Lpl, Pepck, lipin 1*) (unpublished data). In addition, many of their mRNAs are at lower levels in *Bmal1* ^−/−^ (e.g., *Mod1, Pepck, Enolase3, Pgam*) (unpublished data), consistent with a direct role of Bmal1 in their transcription. Thirdly, these metabolic parameters are disrupted in both circadian mutants with the same rank order of potency as the locomotor activity phenotypes *(Bmal1^−/−^ > Clock^mut^)*. Thus, the most parsimonious interpretation is that the observed metabolic deficiencies in the *Bmal1* ^−/−^ and *Clock^mut^* mice are due to their roles in the circadian clock, rather than to “off-clock” effects.

We observed that the impact of HF on glucose homeostasis was apparently to emphasize the role of the molecular clock. Diet has previously been shown to interact with peripheral clocks. Changes in feeding shift the circadian pattern of gene expression in the liver, but not in the master clock in the SCN (Damiola et al. 2000), demonstrating the importance of food as a cue to circadian control. Individual constituents of food could also provide discrete stimuli. For example, glucose alone can induce rhythmic gene expression in isolated fibroblasts (Hirota et al. 2002). Thus, dietary composition, the size and timing of a feed might all be expected to interact differentially with an underlying circadian regulation of metabolic control.

Alterations in dietary content, the availability of “fast food,” inactivity, and sociocultural factors have all been implicated in the emergence of the metabolic syndrome as a major challenge to the public health (Zimmet et al. 2001). However, while mechanistic integration of the diverse elements of the syndrome has proven elusive, our studies suggest that timing may influence the functional consequences of ingesting a caloric load.

## Materials and Methods

### 

#### Animals

Mice were acclimatized for 2 wk in 12 h light–12 h dark cycles before being subjected to a 36-h period of constant darkness followed by experimentation in darkness. Experimental chronology is measured in CT, subjective day beginning at 7 AM (CT0), and subjective night beginning at 7 PM (CT12).

#### Diet

WT and *Clock^mut^* mice were placed on an HF (Teklad, TD02435) and compared to age-matched WT mice on a regular chow diet (RC). Mice were on RC for 8 wk except for those subjected to FSIGT where they received RC for 11 mo. Body mass composition was measured by dual energy X-ray absorptiometry at 10 mo.

#### Intraperitoneal tolerance

Tests were performed as described (Klaman et al. 2000) with a diminution in the glucose bolus (0.1 g/kg).

#### Intravenous glucose tolerance test and minimal modeling

The tolerance test was performed as described (Pacini et al. 2001) in unanesthetized mice, and the minimal model of Bergman et al. (1979) was applied to the data using MINMOD software (Boston et al. 2003). The derived values were S_i_, S_g_, and acute insulin response to glucose, which measures insulin secretion. S_i_ is the ratio of insulin delivery rate to the interstitium to insulin extraction rate from the interstitium. Long-term feeding of HF to WT mice resulted in imperceptibly small insulin sensitivity values. This could be the consequence of impaired delivery of insulin to the interstitium, exacerbated extraction rate, or a combination of both factors. Insulin secretion is derived from area under the insulin curve, above basal, from 0 to 10 min after glucose infusion; and disposition index, which equals the product of insulin sensitivity multiplied by insulin secretion and measures the degree to which insulin sensitivity can be compensated for by elevated insulin secretion (Pacini et al. 2001).

#### Assay methods

Insulin, leptin, corticosterone, and glucagon levels were measured by immunoassays from Crystalchem (Downers Grove, Illinois, United States), ICN Biochemicals (Costa Mesa, California, United States), and Linco Research (St. Charles, Missouri, United States). Plasma glucose was measured by the glucose oxidase method using a glucose analyzer machine for FSIGT and by glucometer for the intraperitoneal tolerance test. PEPCK activity was quantitated by a bioluminescent method (Wimmer 1988).

#### Statistical analysis

The significance of differences amongst the tolerance test curves was assessed by distribution-free two-way ANOVA with a Bonferroni correction. FSIGT data were tested by one-way ANOVA with the Kruskal-Wallis test. Paired Student's t-tests were used to perform comparisons of corticosterone levels before and after insulin injection in *Bmal^−/−^* mice and between WT and *Clock^mut^* mice. Plasma samples for glucagon analysis were pooled and were thus not compared by a formal statistical analysis. Results are presented as mean ± standard error of the mean (SEM), except for the FSIGT data (Table 2), presented as mean ± fractional standard deviation. Differences were considered significant when *p* < 0.05.

## Abbreviations

*Bmal1*^−/−^**: *Bmal* knockout mice
*Clock^mut^*: *Clock* mutant mice
CT[number]: circadian time [designated time point]
FSIGT: frequent sampling intravenous glucose tolerance test
GT: glucose tolerance
HF: high-fat diet
IT: insulin tolerance
PEPCK: phosphoenolpyruvate carboxykinase
RC: regular chow diet
SCN: suprachiasmatic nucleus
S_g_: glucose-mediated glucose disposal
S_i_: insulin sensitivity
WT: wild-type
